# Supplementary material for: Toward Unraveling Cyanopolyyne Surface Chemistry: A Preview on Isolated Systems from HC3N to Ethyl Cyanide and Propylamine
Source: ACS Earth Space Chem. 2025 May 15;9(6):1534–43. doi: 10.1021/acsearthspacechem.5c00021 (PMC12186827; doi:10.1021/acsearthspacechem.5c00021)
Supplement: Supplementary file 1 [file sp5c00021_si_001.pdf]

Supporting Information: Toward Unraveling Cyanopolyyne Surface Chemistry: A Preview on Isolated Systems From HC_3N to Ethyl Cyanide and Propylamine

Marten T. Raaphorst,[*,†] Joan Enrique-Romero,[†] and Thanja Lamberts[*,†,‡]

†*Leiden Institute of Chemistry, Leiden University*

‡*Leiden Observatory, Leiden University*

E-mail: m.t.raaphorst@lic.leidenuniv.nl; a.l.m.lamberts@lic.leidenuniv.nl

1 HC_3N+H benchmark

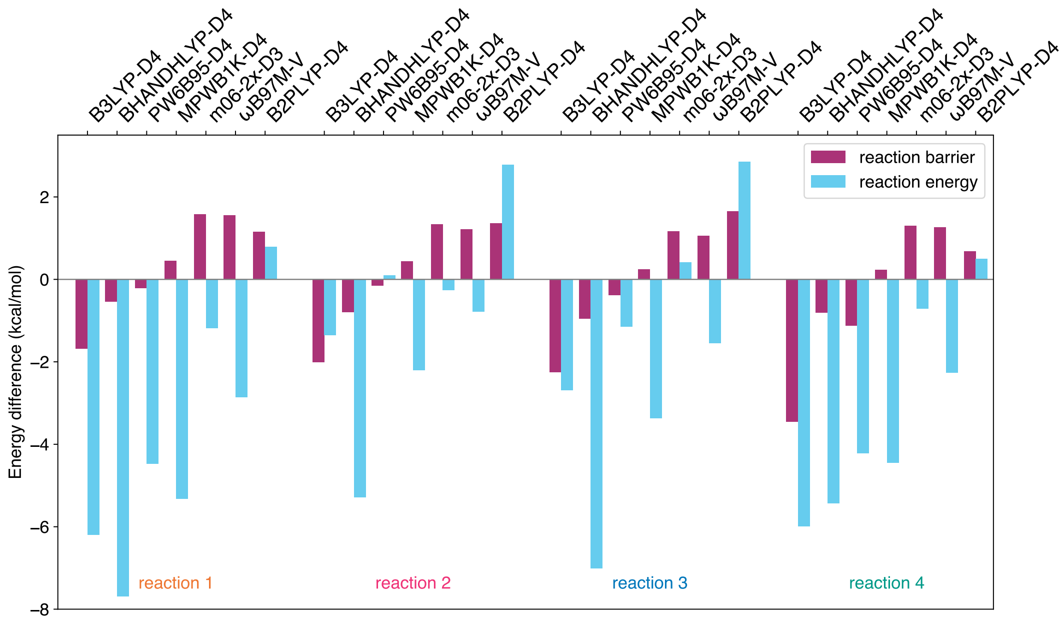

Figure S1: Benchmark results for the HC_3N+H reactions. The energy differences between the values obtained for each density functional (with def2-TZVP basis) and the uCCSD(T)-F12/VTZ-F12 values are plotted. ZPE corrections are not included. The reaction energies and barrier heights are given relative to the prereaction state. The labels reaction 1-4 correspond to addition to C1, C2, C3 and N respectively. All geometries were preoptimized with B3LYP-D4/def2-TZVP and subsequently optimized with the respective density functional, with VeryTightOpt and VeryTightSCF settings. The uCCSD(T)-F12 single point calculations were performed on the B3LYP-D4 geometries. We used the recommendations from the ORCA manual regarding the integration grid, employing the def2grid or def3grid in a consistent way within a given reaction.

2 IRC plots

For the reactions CH_2CHCN+H and $CH_2CHCHNH$+H the energy plots obtained from IRC calculations are given in Figure S2 and Figure S3. On the horizontal axes the intrinsic reaction coordinates are given in units of Bohr $\cdot \sqrt{\text{amu}}$. These coordinates were calculated from all the geometries along the IRC trajectory in the following manner: The mass-weighted intrinsic reaction coordinate q_j for a given geometry j is given by

$$q_j = \sum_i d_{i,j} \cdot \sqrt{m_i}, \tag{1}$$

where m_i is the mass of atom i and $d_{i,j}$ is the displacement of atom i in geometry j relative to the TS calculated with

$$d_{i,j} = \sqrt{(x_{i,j} - x_{i,TS})^2 + (y_{i,j} - y_{i,TS})^2 + (z_{i,j} - z_{i,TS})^2}, \tag{2}$$

with $x_{i,TS}, y_{i,TS}$ and $z_{i,TS}$ the coordinates of atom i in the TS geometry. More information on calculating the mass-weighted intrinsic reaction coordinate can be found in the paper by Deng and Ziegler[S1].

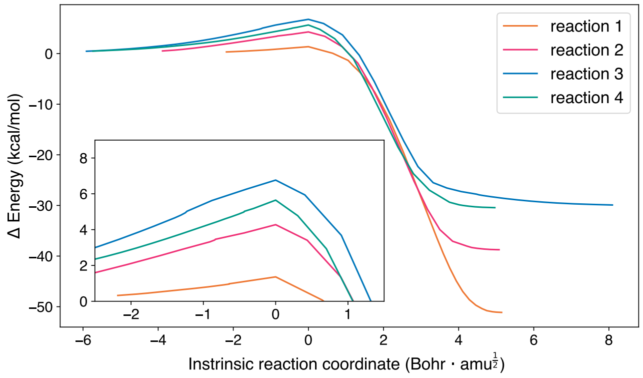

Figure S2: Energy plot of the IRC calculations for the CH_2CHCN+H addition reactions. The IRC calculations were performed at the MPWB1K-D4/def2-TZVP level. The labels reaction 1-4 correspond to addition to C1, C2, C3 and N respectively.

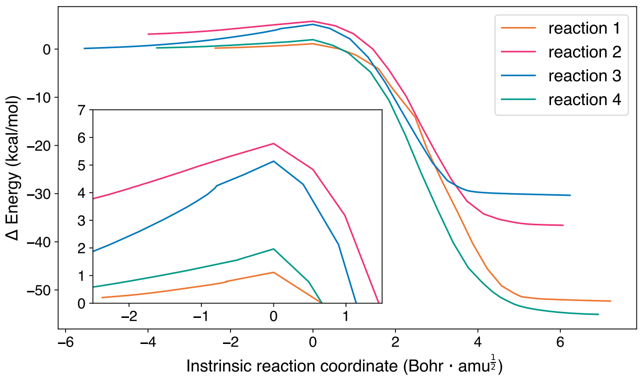

Figure S3: Energy plot of the IRC calculations for the $CH_2CHCHNH$+H addition reactions. The IRC calculations were performed at the MPWB1K-D4/def2-TZVP level. The labels reaction 1-4 correspond to addition to C1, C2, C3 and N respectively.

3 Broken symmetry optimization H_2C_3N+H

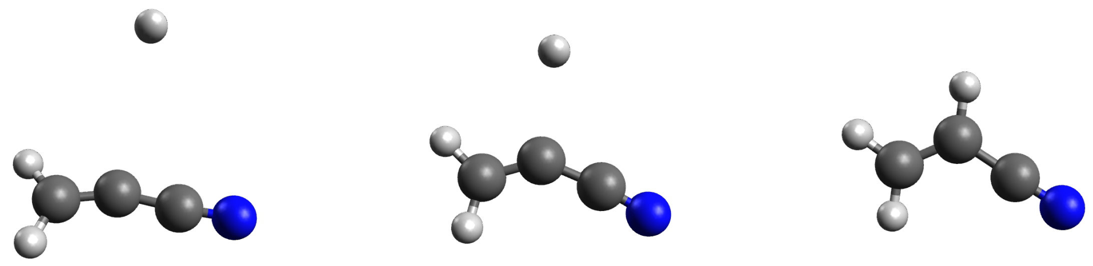

(a) E = -0.1 kcal/mol (b) E = -7.9 kcal/mol (c) E = -108.4 kcal/mol

Figure S4: Snapshots taken along the broken symmetry optimization trajectory, namely, before the reaction (S4a), during the reaction (S4b) and after the reaction (S4c). The calculation was performed with B3LYP-D4/def2-TZVP. Energies given relative to the asymptotic state.

4 MP2 versus DFT for vinyl cyanide + H

We performed geometry optimizations for the H addition to the C1 carbon of vinyl cyanide both using DFT and MP2. These calculations resulted in different transition state geometries and activation energies. Using the same level of theory (rCCSD(T)/aug-cc-pVTZ//MP2/aug-cc-pVTZ) we reproduced the activation and reaction energies reported by Krim et al, confirming that we have found the same saddle point. However, when performing a scan at the uCCSD(T)-F12 level based on the MP2 IRC calculation, we found that the MP2 saddle point is not the real transition state, as can be seen in figure S5a. The dot indicates the geometry of the TS found with the MP2 geometry optimization. However, this point is not the highest point in energy along the reaction coordinate (at the uCCSD(T)-F12 level). The actual highest point is indicated by the X. This implies that MP2 did not find the actual TS. On the other hand, figure S5b shows that the TS found with DFT, indicated with the X, is also the highest point in energy at the uCCSD(T)-F12 level, confirming that this is a correct TS. It should be noted that for both the MP2 and DFT scan, the uCCSD(T)-F12 calculations on a few geometries "after" the TS relative to the reaction path (including the supposed MP2 TS) had a T1 diagnostic of 0.02-0.03, indicating a slight multi-reference character for these geometries.

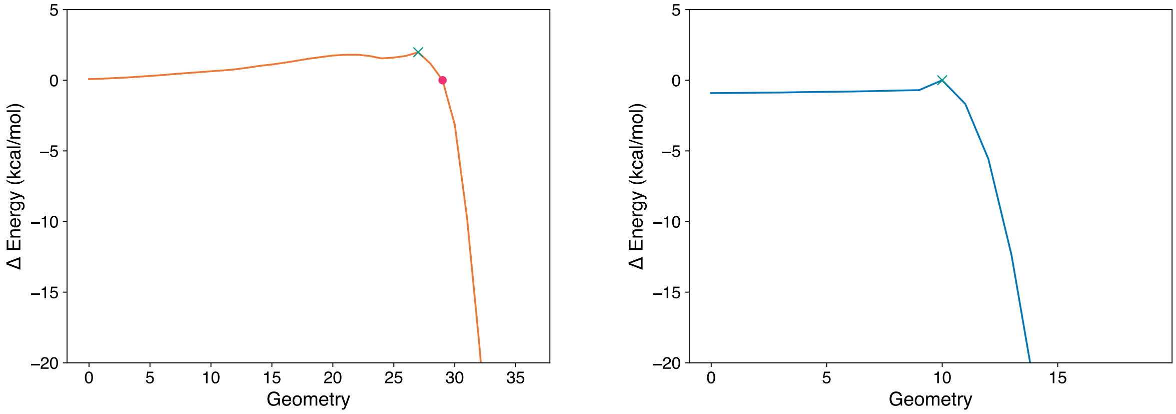

(a) (b)

Figure S5: Scan at the uCCSD(T)-F12 level performed on selected IRC geometries obtained with MP2/aug-cc-pVTZ (S5a) or DFT (MPWB1K-D4/def2-TZVP) (S5b). Energies obtained with uCCSD(T)-F12/cc-pVTZ-F12 are plotted relative to the energy of the supposed TS.

5 Energetics HC_3N hydrogenation network

The full overview of all the reactions studied in this work can be found in Table S1.

References

(S1) Deng, L.; Ziegler, T. The Determination of Intrinsic Reaction Coordinates by Density Functional Theory. *Int. J. Quantum Chem.* **1994**, *52*, 731–765.

Table S1: Energies and cross-over temperatures of all the reactions studied in this work. Reaction energies and barriers in kcal/mol at uCCSD(T)-F12/VTZ-F12//MPWB1K-D4/def2-TZVP level. ZPE corrections in parentheses at the MPWB1K-D4/def2-TZVP level. The labels aC1, aC2, aC3 and aN refer to H addition to the C1, C2, C3 and N atoms respectively.

reaction name	label	reaction energy (kcal/mol)	barrier height (kcal/mol)	T_c (K)
HC_3N + H	aC1	-46.9 (5.0)	3.1 (-0.1)	168
	aC2	-41.4 (5.8)	6.1 (0.0)	218
	aC3	-28.4 (5.3)	7.5 (0.1)	239
	aN	-26.8 (4.8)	6.6 (-0.2)	250
CH_2CHCN + H	aC1	-48.0 (5.8)	1.5 (0.7)	118
	aC2	-37.3 (5.1)	4.2 (0.9)	201
	aC3	-27.1 (6.1)	6.6 (0.9)	218
	aN	-24.5 (5.3)	6.5 (0.5)	251
CH_3CH_2CN + H	aC3	-27.2 (6.0)	5.6 (0.9)	210
	aN	-22.0 (6.3)	8.5 (0.5)	273
CH_2CCNH + H	aC1	-56.9 (6.3)	0.8 (0.7)	102
	aC2	-58.7 (6.9)	1.2 (0.9)	109
	aC3	-54.1 (6.7)	1.6 (1.0)	159
	aN	-52.9 (6.3)	2.7 (0.8)	179
CH_3CCNH_2 + H	aC2	-47.4 (6.7)	1.6 (0.9)	119
	aC3	-43.9 (6.8)	2.3 (1.0)	158
$CH_2CHCHNH$ + H	aC1	-49.2 (5.9)	1.2 (0.6)	115
	aC2	-34.5 (5.1)	5.6 (0.8)	170
	aC3	-28.3 (5.6)	5.2 (0.9)	218
	aN	-50.7 (6.1)	2.1 (0.7)	150
CH_3CHCNH + H	aC2	-49.0 (7.1)	2.0 (1.1)	143
	aC3	-56.9 (6.9)	2.8 (1.0)	192
	aN	-36.5 (7.0)	5.4 (1.1)	242
$CH_3CHCHNH_2$ + H	aC2	-41.7 (6.5)	0.2 (0.9)	71
	aC3	-34.4 (5.8)	3.5 (0.9)	196
$CH_2CHCH_2NH_2$ + H	aC1	-40.3 (5.4)	1.5 (0.7)	118
	aC2	-38.1 (5.5)	3.3 (1.1)	177
CH_3CH_2CHNH + H	aC3	-31.5 (5.6)	4.5 (0.9)	185
	aN	-39.6 (6.3)	3.6 (1.0)	163
